# The Extent of Edgetic Perturbations in the Human Interactome Caused by Population-Specific Mutations

**DOI:** 10.3390/biom14010040

**Published:** 2023-12-27

**Authors:** Hongzhu Cui, Suhas Srinivasan, Ziyang Gao, Dmitry Korkin

**Affiliations:** 1 Bioinformatics and Computational Biology Program, Worcester Polytechnic Institute, Worcester, MA 01609, USA; zgao@wpi.edu; 2 Chromatography and Mass Spectrometry Division, Thermo Fisher Scientific, San Jose, CA 95134, USA; 3 Data Science Program, Worcester Polytechnic Institute, Worcester, MA 01609, USA; suhas.srinivasan@stanford.edu; 4 Program in Epithelial Biology, Stanford School of Medicine, Stanford, CA 94305, USA; 5 Center for Personal Dynamic Regulomes, Stanford School of Medicine, Stanford, CA 94305, USA; 6 Computer Science Department, Worcester Polytechnic Institute, Worcester, MA 01609, USA

**Keywords:** population genetics, functional nsSNVs, protein-protein interaction, interactome, edgetic, non-synonymous mutations

## Abstract

Until recently, efforts in population genetics have been focused primarily on people of European ancestry. To attenuate this bias, global population studies, such as the 1000 Genomes Project, have revealed differences in genetic variation across ethnic groups. How many of these differences can be attributed to population-specific traits? To answer this question, the mutation data must be linked with functional outcomes. A new “edgotype” concept has been proposed, which emphasizes the interaction-specific, “edgetic”, perturbations caused by mutations in the interacting proteins. In this work, we performed systematic in silico edgetic profiling of ~50,000 non-synonymous SNVs (nsSNVs) from the 1000 Genomes Project by leveraging our semi-supervised learning approach SNP-IN tool on a comprehensive set of over 10,000 protein interaction complexes. We interrogated the functional roles of the variants and their impact on the human interactome and compared the results with the pathogenic variants disrupting PPIs in the same interactome. Our results demonstrated that a considerable number of nsSNVs from healthy populations could rewire the interactome. We also showed that the proteins enriched with interaction-disrupting mutations were associated with diverse functions and had implications in a broad spectrum of diseases. Further analysis indicated that distinct gene edgetic profiles among major populations could shed light on the molecular mechanisms behind the population phenotypic variances. Finally, the network analysis revealed that the disease-associated modules surprisingly harbored a higher density of interaction-disrupting mutations from healthy populations. The variation in the cumulative network damage within these modules could potentially account for the observed disparities in disease susceptibility, which are distinctly specific to certain populations. Our work demonstrates the feasibility of a large-scale in silico edgetic study, and reveals insights into the orchestrated play of population-specific mutations in the human interactome.

## 1. Introduction

Since the completion of the Human Genome Project, scientists have made remarkable advancements in high-throughput genotyping technology, in particular in next-generation sequencing technology (NGS) [1,2,3]. This achievements have been made alongside large-consortium sequencing efforts, such as the International HapMap project [4] and the 1000 Genomes Project [5]; genotyping hundreds to thousands of individuals has become a common practice. Large-consortium sequencing projects have revealed that many genetic variations are population-specific [5,6]. Genetic differences among the population datasets have been critical in interpreting the phenotypic differences between populations, and they have important implications for human health and diseases. Despite their potential significance, population-specific single-nucleotide polymorphisms (SNPs) have received limited attention, and are not yet commonly considered in clinical practice.

Along with the development of NGS technologies, genetic variation databases have been developed to help scientists make sense of the vast amount of NGS data, including the 1000 Genomes Project [7] and Single-Nucleotide Polymorphism Database (dbSNP) [8]. Because many genetic variants have not been previously described, the role of computational approaches in functional annotation has become increasingly important [9,10]. There are many bioinformatics tools for the functional annotation of genetic variations. Several recent reviews [11,12,13,14] give a comprehensive survey of state-of-the-art variant annotation methods. Most of the tools are either sequence-based or evolutionary conservation-based, focusing on the annotation of single-nucleotide variants (SNVs) because they are easier to capture and analyze [10]. If an SNV can be mapped on the experimentally determined protein structure or a corresponding accurate homology model, then one can compute some properties using the structure information, which could, in turn, improve the accuracy of predicting the functional impact of this mutation [15]. Recently, our group developed a new computational method [16] called the SNP-IN tool (non-synonymous SNP INteraction effect prediction tool) [16] to predict the effects of non-synonymous SNVs (nsSNVs) on protein–protein interactions (PPIs), given these interactions’ experimental structure or structural model. The method leverages supervised and semi-supervised learning models, including a new random forest self-learning protocol. The accurate and balanced performance of the SNP-IN tool allows for the proteome-scale functional annotation of non-synonymous SNVs.

At the same time, network biology has become an important approach for in silico analyses, with the goal of investigating the organizing principles of intra-cellular networks and the implications of these principles for understanding diseases [17]. Furthermore, a complex phenotype is rarely a consequence of a single genetic mutation; it is usually the effect of genotype–environment (GxE) interaction. This complexity is further increased by the physical interactions of the genetic factors in the context of a biological network [18], with the biological networks being a natural framework for integrating different sources of data and incorporating prior knowledge [19]. Among various types of biological networks, the protein–protein interaction (PPI) network, or interactome, has attracted the most attention and is arguably the most widely studied network in biology [17,20]. 

Recently, the concept of the “edgotype” was proposed for PPI networks [21], which described the functional outcomes of genetic variants on PPIs, and the corresponding rewiring effects on the interactome. In contrast to a traditional, genotype-based, view wherein a genetic variation may or may not cause the loss of a protein function, the edgetic perturbation model describes variation as interaction-specific, i.e., the genetic variation may or may not cause the removal or addition of a specific PPI, while other PPIs remain unperturbed. Recent studies have shown that different mutations lead to different defects in proteins, and this may cause distinct perturbations in the protein–protein interaction network [22,23,24]. However, profiling thousands of missense mutations experimentally using interaction assays remains a costly and labor-intensive task. For the edgetic perturbation model, our SNP-IN tool can be used to carry out in silico edgetic profiling.

Edgetics is a novel approach to understanding genotype–phenotype relationships in the context of the biological network. The edgotype provides the alternative, mechanistic, explanation of a mutation’s functional impact, thus dissecting many complex genotype–phenotype relationships [22]. An edgetic alteration can cause the removal of one or several interactions, while leaving the rest intact and functional. The alteration might have a more subtle impact on the network, not necessarily resulting in a disease phenotype [25]. More importantly, the edgetic perturbation model can often easily explain confounding genetic phenomena, such as genetic heterogeneity [26,27]. Edgetics provides us with a similar recipe to study population genetics. With the amounts of genetic variation data from different populations around the world contained in global consortiums such as the 1000 Genomes Project [7,28] and ENCODE [29], we have a unique opportunity to move beyond traditional population genetics, and adopt a “population edgetics” approach to studying genetic differences within and between populations. We also expect that the distinct edgetic profiles—rather than the mutation data alone—can better explain why there are different disease frequency patterns and susceptibilities across different populations. Ultimately, network-based analysis of the genetic architectures may not only shed light on the biological mechanisms underlying complex phenotypes, but may also yield better ways of measuring genetic predisposition to a certain disease in healthy individuals.

In this work, we created a comprehensive catalog of population-specific edgetic effects at the whole-interactome level using population genetics data. Specifically, our recently developed SNP-IN tool was applied to 46,599 nsSNVs collected from the 1000 Genomes Project dataset, by leveraging the structural information on PPI complexes obtained either experimentally or through modeling. We predicted that a considerable amount of healthy population-specific nsSNVs could rewire PPIs. We also showed that the proteins (and the corresponding genes) enriched with the interaction-disrupting mutations obtained from healthy populations were, in fact, associated with diverse functions and implicated in various diseases. Our analysis indicated that some gene edgetic profiles were distinct from each other across the five major populations, and could help explain the population phenotypic variance. Finally, the network analysis reveals phenotype-associated modules heavily laden with the interaction-disrupting mutations. The observable discrepancy in the accumulated damage within these modules potentially indicates a variation in disease susceptibility specific to certain populations, warranting further investigation.

## 2. Materials and Methods

### 2.1. Genetic Mutation Data Processing and Construction of the Human Interactome

The mutation data are collected from the 1000 Genome Project [5], a major international consortium effort to catalog genetic variants across major ethnic populations. In the functional annotation and downstream analysis, we focus exclusively on non-synonymous missense single-nucleotide variants (nsSNVs) and excluded non-coding variations, synonymous SNVs, short indels, and structural variations. A single-nucleotide variant is the simplest and yet the most common type of genetic variation among people. In this work, nsSNVs are selected because they are most likely to make a functional impact on the PPIs. The mutation data are first processed with ANNOVAR [30] to obtain SNV locations on the genes and their protein products and determine the corresponding residue substitution. This information is essential for the SNP-IN tool [16] to predict the rewiring effects of mutations. 

To construct a unified human interactome, we use two large-scale protein–protein interaction data sources: the High-quality INTeractomes database (HINT) [28], and the Human Reference Protein Interactome Mapping Project (HuRI) [29]. HINT is a centralized database of human PPIs integrated from several other sources and annotated using both an automated protocol and manual curation. HuRI focuses on the experimentally validated PPIs using yeast two-hybrid experiments. The two PPI sources are merged because they provide complementary views of the whole human interactome. The HINT database contains 63,684 interactions, while the HuRI dataset includes 76,537 interactions. When merged, the final human interactome includes 105,087 interactions, with 35,134 interactions occurring in both datasets. 

### 2.2. Functional Annotation of nsSNV

Next, we determine if a mutation occurring in a protein can affect the protein–protein interaction that this protein is involved in. To do so, consider two PPIs: a wild-type interaction, which involves a wild-type version of that protein, and a mutant PPI, which involves the protein with the mutation. To accurately determine the functional damage to a PPI caused by a mutation, our approach requires information about both the mutation and the PPI structure or structural model as the input for the SNP-IN tool [16,31]. The prediction task by the SNP-IN tool is then formulated as a classification problem, with three classes of rewiring effects: beneficial, neutral, and detrimental. The effects are assigned based on the difference between the binding free energies, ΔΔG, of the mutant and wild-type PPIs. Specifically,

ΔΔG=ΔGmt−ΔGwt,

where ΔGmt and ΔGwt are the mutant and wild-type binding free energies, respectively. The beneficial, neutral, or detrimental types of mutations are then determined by applying two previously established thresholds to the ΔΔG values [32,33]:

$$\mathrm{Beneficial}:\;\Delta \Delta G<-0.5\frac{\mathrm{kcal}}{\mathrm{mol}}$$

$$\mathrm{Neutral}:-0.5\frac{\mathrm{kcal}}{\mathrm{mol}}\leq \Delta \Delta G<0.5\frac{\mathrm{kcal}}{\mathrm{mol}}$$

Detrimental: ΔΔG≥0.5 kcal/mol

A PPI structure for the SNP-IN tool is obtained in two ways. First, if a PPI has a native structure of the corresponding protein complex, we extract it from the Protein Data Bank [34]. If there is no native structure for a PPI, we apply homology, or comparative modeling [35] to obtain a structural model, either for the full-length PPI complex or at least for a pair of protein domains that form the interaction interface [16,31]. Lastly, we define a disruptive gene as a gene carrying at least one detrimental mutation, which causes a disruptive effect on the related protein–protein interactions based on SNP-IN tool output. 

### 2.3. dN/dS Ratio Calculation and Comparison

A high-confidence collection of cancer genes was obtained by merging the Cancer Gene Census dataset [36] and a recently published computationally predicted cancer gene set, MutPanning [37]. The Catalogue of Somatic Mutations in Cancer (COSMIC) and Cancer Gene Census (CGC) are ongoing efforts to catalog genes with known mutations that have been causally implicated in cancer. CGC is used as a golden standard in cancer genetics [36], with each driver gene being accompanied by an expert-curated description. MutPanning is a computational method for the identification of cancer driver genes across 28 tumor types that combines the characteristic contexts around passenger mutations with the signals of mutational recurrence [37]. The two cancer driver gene sources contain 723 and 460 genes, respectively, with an overlap of 196 genes. In total, 987 distinct cancer genes are collected. Next, the list of 3804 housekeeping genes is retrieved from a dataset compiled by Eisenberg and Lavanon [38]. Housekeeping genes are the genes that are essential for the existence of a cell and the maintenance of the basal cellular functions [39]. The genes are believed to express in all cells of an organism under normal and pathophysiological conditions [40]. 

The evolutionary rates (ER) of the genes are calculated using a ratio between the number of non-synonymous substitutions (dN) to the number of synonymous substitutions, dN/dS [41]. We calculate the dN/dS ratio by comparing the human gene sequences with the orthologous groups. Specifically, the orthologous genes of macaque, gorilla, orangutan, chimpanzee, and gibbon corresponding to the human genes under consideration (GRCh38.p13) are queried from the Ensembl Biomart platform [42], together with the corresponding dN and dS values. The dN/dS ratio is then calculated, while the missing and infinite values are removed. For sets of homologs that do not include exactly one representative in each organism, the group mean of ER is calculated as the representative. The overall ER of a human gene is the average value of ERs for an individual organism, and we only include genes with orthologs in more than three organisms. The comparison of ER between different sets of genes is made with a Wilcoxon test, because no prior information about the underlying distribution is known.

### 2.4. Calculation of Disruptive Mutation Rate in Proteome and GO Enrichment Analysis

The disruptive mutation rate of a gene is the ratio of the total number of detrimental mutations in a corresponding protein to the protein’s length. We first collect all detrimental mutations occurring in the protein based on our SNP-IN tool annotation. The protein sequence length information is retrieved from Uniprot [43]. The average disruptive mutation rate across the proteome is considered as the background rate, and we define proteins (and the corresponding genes) with the disruptive mutation rate greater than the average rate plus a standard deviation to be enriched with the detrimental mutations. 

We then investigate the biological implications of genes enriched with detrimental mutations using gene ontology (GO) [44]. For the set of genes enriched with detrimental mutations, a GO enrichment analysis is carried out to obtain a list of enriched GO terms. In the analysis, we use the third level of the GO hierarchy and consider only those GO terms with *p*-value ≤ 0.01. By comparing the results from the second and fourth levels, we find that the third level represents the best trade-off between having too general (and thus not very informative) but well-populated GO terms from the second level, and more specific but not well-populated (and thus not suitable for the enrichment analysis) terms from the fourth level. The GO enrichment is performed using DAVID [45], and multiple testing correction is carried out with false discovery rate estimation [46].

### 2.5. Population-Specific Edgetic Profiles of Genes Enriched with Detrimental Mutations

For a protein (and the corresponding gene) enriched with detrimental mutations, we introduce the population edgetic profile concept to describe the diverse network rewiring effects caused by detrimental mutations centering around the mutated gene across different populations. Simply put, the edgetic profile, $EP\left(p\right),$ of a protein p that participates in m interactions is represented as a sequence of vectors $\left(v_{1},\;v_{2},\ldots ,v_{m}\right)$ of different lengths, $l_{1},\;l_{2},\ldots ,l_{m}$ . Each vector $v_{i}$ consists of a list of $l_{i}$ detrimental mutations targeting the same interaction i . The element takes only binary values, where 1 indicates a detrimental mutation with a non-zero allele frequency for a specific population, and 0 indicates a detrimental mutation that is not present in this population. For example, $EP\left(p\right)=\left(\left[1,\;0,\;0,\;1\right],\;\left[0,\;1,\;1\right],\;\left[1,\;0,\;0,\;1,\;1\right]\right)$ corresponds to an edgetic profile of a protein that has three interaction partners. 

To compare the edgetic profiles, $EP^{\left(i\right)}\;\mathrm{and}\;EP^{\left(j\right)},$ of the same protein in two different populations, i and j , respectively, we first calculate the Manhattan distance between two profile vectors after merging the original sequence of vectors into a single vector of dimension:

$$l=\sum_{k=1}^{m}l_{k}:\;d\left(EP^{\left(i\right)},\;EP^{\left(j\right)}\right)=||EP^{\left(i\right)}-EP^{\left(j\right)}{\left|\right|}_{1}=\sum_{k=1}^{l}\left|EP_{k}^{\left(i\right)}-EP_{k}^{\left(j\right)}\;\right|$$

The Manhattan distance between two edgetic profiles is further normalized by taking the total number of detrimental mutations into account:

$$d_{\mathrm{norm}}\left(EP^{\left(i\right)},\;EP^{\left(j\right)}\right)=\frac{d\left(EP^{\left(i\right)},\;EP^{\left(j\right)}\right)}{l}$$

The average difference of gene edgetic profiles between any two populations is then evaluated as follows:

$$d\left(EP^{\left(i\right)},\;EP^{\left(j\right)}\right)=\frac{\sum_{i=1}^{N}\sum_{j=i+1}^{N}d_{\mathrm{norm}}\left(EP^{\left(i\right)},\;EP^{\left(j\right)}\right)}{N\left(N-1\right)}$$

To assess the statistical significance of the final average distance for the 1000 Genomes Project dataset, we randomly generate the edgetic profiles for each major population. Specifically, given an edgetic profile for one population, we randomly shuffle the list of values in that profile. With such randomly shuffled edgetic profiles, we further calculate the average difference following the steps described above. We repeat such randomized experiments 1000 times to generate the sample population for assessing the statistical significance. Z-score and the corresponding *p*-value are calculated to determine the significance of our dataset. 

### 2.6. Examination of Topological Properties of Genes Enriched in Detrimental Mutations in Human Interactome

In our comparative network analysis, we independently investigate and compare the topological importance of the rewired edges targeted first by the pathogenic mutations and then by the healthy population mutations. To quantify the importance, we apply two edge centrality measures to the network: shortest-path edge betweenness and current-flow betweenness. Betweenness centrality was proposed as a general measure of centrality [47]. It was applied to a wide range of real-world problems, including problems related to biological [48], social [49], and transportation networks [50]. Typically, an edge with a higher betweenness centrality in a complex network will be more critical for the network, because more information will pass through that edge. In the human interactome, mutations disrupting an interaction corresponding to such an edge are likely to have a greater impact on the cell functioning. The shortest path edge betweenness centrality is a measure of centrality based on the number of the shortest paths that go through a given edge. The measure is defined as the sum of the fractions of all-pair shortest paths passing through an edge [51]. Formally, the shortest path edge betweenness centrality of an edge e is given by following expression:

$$c_{B}\left(e\right)=\underset{u,v\;\in V}{\sum }\frac{\sigma \left(u,v|e\right)}{\sigma \left(u,v\right)}$$

where V is the set of nodes, $\sigma \left(u,v\right)$ is the number of shortest paths between a pair of nodes, u and v , and $\sigma \left(u,v|e\right)$ is the number of those paths passing through edge e . The current-flow betweenness is another global centrality measure, which is based on an electrical current model for information dispersion. It is also known as random-walk betweenness centrality [52]. 

Another graph-based metric to characterize the rewiring effect caused by detrimental mutations is the network efficiency [17]. The concept of efficiency measures how well the network propagates and exchanges information. To compute the efficiency of the subnetworks targeted by pathogenic mutations and normal mutations, we first constructed two separate subnetworks by grouping the corresponding disrupted interactions. For a pair of nodes in the network, the efficiency is the multiplicative inverse of the shortest path distance between the pair. The global efficiency of a graph is defined as the average efficiency of all pairs of nodes. Formally, it is defined as

$$E\left(G\right)=\frac{1}{N\left(N-1\right)}\;\underset{i\neq j\;\in G}{\sum }\frac{1}{d\left(i,j\right)}$$

where N is the number of nodes in a network and $d\left(i,j\right)$ is the length of the shortest path between a pair of nodes, i and j . 

### 2.7. Phenotype-Associated Community Detection in the Human Interactome and Its Enrichment with Detrimental Mutations

Discovering biologically relevant modules is a challenging task [53,54]. Methods for module discovery primarily come in two forms. Methods of the first type identify the modules in a biological network using exclusively the information on the network’s topology. The main challenge for such methods is the lack of any relevant biological information about the proteins constituting the network. Methods of the second type initiate the module with the “seed” genes or proteins, and then gradually grow the module by attaching additional genes/proteins from the network. In this work, we explore methods of both types.

For the first type, we adopt a technique based on the idea of “diffusion state distance” (DSD) [55], which has been proven to be the best performer in the DREAM Module Identification challenge [56], as it yields the highest number of the phenotype-associated modules. The approach has two main steps: computing a DSD matrix and applying spectral clustering on the DSD matrix to identify phenotype-associated modules. As a proximity measure, DSD is conceptually different from the traditional shortest-path measure: the latter favors the hub-like nodes in the network and does not incorporate information about the intrinsic network structure. Intuitively, a protein pair connected by paths through low-degree nodes share more functional similarity than other protein pairs connected by paths that go through hubs. (See Supplementary Figure S4). So, DSD is a more fine-grained measure of similarity that “downweighs” the hubs in the human interactome. Formally, given an undirected graph $G\left(V,\;E\right)$ consisting of a node set $V=\left\{v_{1},v_{2},\ldots ,v_{n}\right\}$ and $\left|V\right|=n$ , we define a vector $D^{k}\left(A\right)$ :

$$D^{k}\left(A\right)=(D^{k}\left(A,\;v_{1}\right),\;D^{k}\left(A,\;v_{2}\right),\;\ldots ,\;D^{k}\left(A,\;v_{n}\right)),$$

where $\;D^{k}\left(A,\;v_{i}\right)$ is the expected number of times that a simple symmetric random walk starting at node A , and proceeding for k steps, will visit node $v_{i}$ . Thus, $D^{k}\left(A\right)$ defines a global distance measure from node A to all the other nodes of the network. Assume k is fixed. Then, the DSD between nodes A and B is defined as follows:

$$\mathrm{DSD}\left(A,\;B\right)=||D^{k}\left(A\right)-D^{k}\left(B\right){\left|\right|}_{1}$$

The DSD matrix is calculated using the “cDSD” method [57] from software available at: http://dsd.cs.tufts.edu/capdsd accessed on 10 August 2023. The follow-up spectral clustering on the DSD matrix is performed with scikit-learn package [58]. 

For the second type, we consider a recently published method, DIseAse MOdule Detection (DIAMOnD) [59]. DIAMOnD is a module detection algorithm that utilizes known seed genes to identify disease modules by adding new proteins according to their connection significance to the seed proteins. The algorithm exploits the fact that the disease-associated proteins do not reside within locally dense communities, and thus the significance of their connections would be a more predictive measure when compared to the local network density. In addition, the use of the significance of the number of connections reduces spurious detection of high-degree proteins, compared to using the absolute number of connections. As a result, DIAMOnD produces a connected disease module together with a list of disease-associated protein candidates ranked by the connectivity significance.

## 3. Results

**Figure 1:**
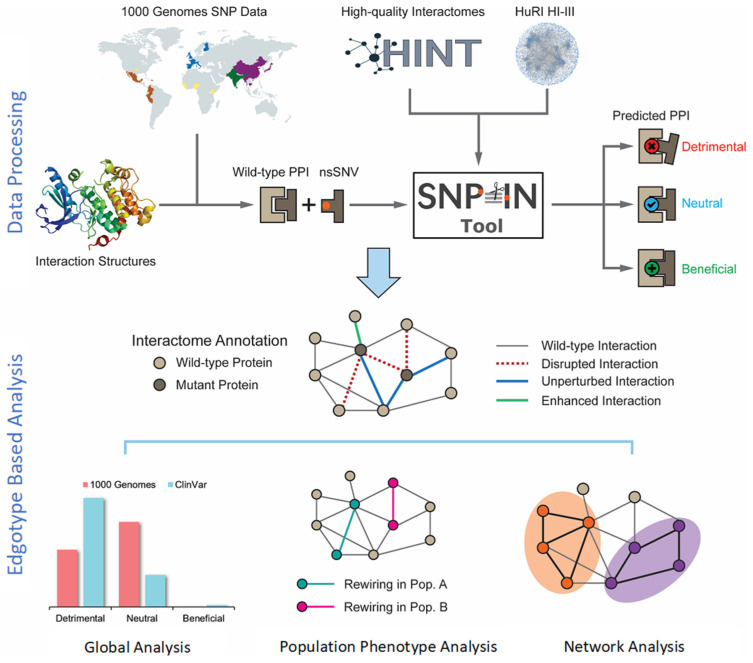
Overview of the integrated computational workflow. The workflow integrated biological data of three different kinds: population variation (mutations), protein structure, and protein interaction network. The population mutation data are collected from the 1000 Genomes Project. Then, the mutations are mapped to structurally resolved protein–protein interaction complexes, and the SNP-IN tool is used for functional annotation. The analysis stage includes global evaluation of rewiring effects by mutations, edgotype-based analysis of population phenotypic variance, and network analysis of detrimental mutations.

A systematic analysis of the effects of population-specific mutations on the human interactome was conducted using the SNP-IN Tool [16] (Figure 1). We first collected and processed population-specific mutation data from the 1000 Genomes Project (https://www.internationalgenome.org/ accessed on 10 August 2023). After that, the population-specific nsSNVs were mapped onto the structures of protein–protein interaction complexes. This structural information was necessary for the SNP-IN tool’s input to predict the mutation’s effect on the corresponding protein–protein interaction (PPI). Specifically, the SNP-IN tool annotated the impact of an nsSNV on a PPI as *neutral* (no change in the PPI), *detrimental* (loss of the PPI due to decreased binding affinity), or *beneficial* (substantially increased binding affinity of the PPI). With the functional characterization provided by the SNP-IN tool, we then evaluated interactome-wide mapping of the mutations followed by large-scale edgetic profiling of the proteins enriched with the detrimental mutations. Based on the concept of edgotype, we investigated the potential role of the human interactome’s rewiring, caused by population-specific detrimental mutations, and the resulting phenotypic variance across the populations. Finally, we leveraged the network analysis and functional module enrichment techniques to deepen our understanding of the impact of these detrimental mutations on the human interactome. 

### 3.1. Interaction-Disrupting nsSNVs Have a Relatively Abundant Presence in Healthy Populations and Unique Evolutionary Traits 

**Figure 2:**
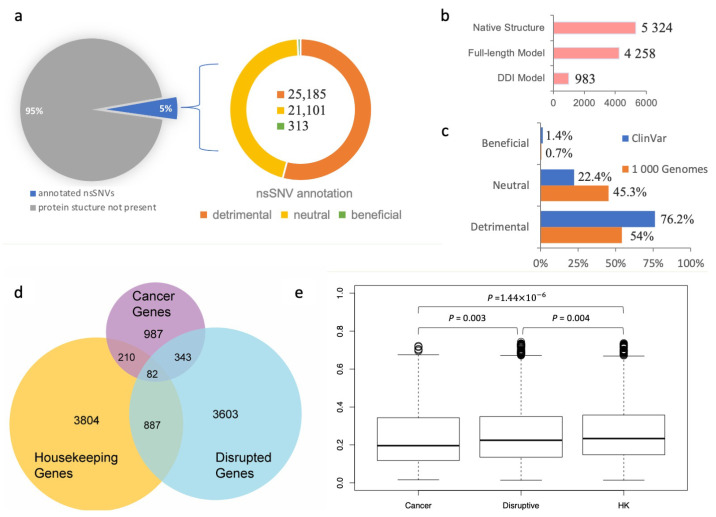
Analysis of population-specific nsSNVs annotated using the SNP-IN tool. (**a**) SNP-IN tool annotation covers about 5% of total non-synonymous SNVs in the 1000 Genomes Project; all three type of effects on PPIs were detected, with detrimental nsSNVs being the dominant type and beneficial nsSNVs contributing a substantially smaller fraction of variants than the other two types. (**b**) Three sources for the structural data on protein interaction complexes: native PPI structure, full-length PPI model, and domain–domain interaction (DDI) model. Overall, 46,599 nsSNVs mapped to 10,565 protein interaction complex structures were annotated; multiple nsSNVs were annotated with more than one source. (**c**) Comparison of SNP-IN tool-based annotations of pathogenic mutations from ClinVar database and population-specific mutations from the 1000 Genomes Project. As expected, the population-specific nsSNVs obtained from the healthy population include a significantly larger proportion of neutral mutations compared to pathogenic nsSNVs. (**d**) Three sets of genes used in our dN/dS analysis and their mutual overlap: disruptive genes, cancer genes, and housekeeping (HK) genes. (**e**) Comparison of dN/dS ratios across three gene sets.

Genetic variation data were extracted from the 1000 Genomes Project dataset of more than 88 million genetic variants [5]. The majority of the variants were SNVs (84.7 million); however, the dataset also included ~3.6 million short indels and ~60,000 structural variants. In this work, we focused on 513,149 non-synonymous SNVs (nsSNVs), for which the corresponding residue substitution on the protein products was determined. The nsSNVs were then mapped either to experimentally obtained protein–protein interaction structures or their structural models (see Section 2 for more detail). Overall, 32,186 nsSNVs were mapped to 5324 native protein–protein interaction structures, 18,119 nsSNVs to 4258 full-length protein–protein interaction models, and 3790 nsSNVs to 983 domain–domain interaction models (Figure 2b). Given that a single nsSNV may be mapped to multiple experimental or computationally modeled protein–protein interaction interfaces, in total, the SNP-IN tool annotated 46,599 population-specific nsSNVs based on the aforementioned mappings.

Our results showed that among the 46,599 population-specific nsSNVs (5% of all collected nsSNVs) (Figure 2a), 25,185 nsSNVs (54.1% of annotated nsSNVs) were predicted detrimental to at least one PPI involving the corresponding mutant protein, and only 313 nsSNVs (0.7% of annotated nsSNVs) were labeled beneficial to a PPI (see Figure 2c). In our previous work, we applied the same in silico protocol to annotate a comprehensive set of 3401 pathogenic mutations occurring in the genes associated with human diseases extracted from the ClinVar database (https://www.ncbi.nlm.nih.gov/clinvar/ accessed on 10 August 2023) [60]. We found that 2592 nsSNVs (76.2%) were predicted detrimental to at least one PPI, and 48 nsSNVs (1.4%) were predicted beneficial (Figure 2c). Our results showed that the proportion of the detrimental SNVs obtained from the 1000 Genomes Project dataset, and thus observed in the healthy population, was 1.5 times smaller, but there was still a substantial number of nsSNVs that were expected to alter protein–protein interaction. Interestingly, both datasets of mutations shared significantly smaller fractions of the beneficial mutations, supporting our previous observation that genetic variations enhancing the protein–protein interaction were rare in the human genome [31]. An nsSNV predicted detrimental is likely to be functional too, since it disrupts a PPI and substantially affects the molecular function associated with it. If the loss of function further increases an individual’s susceptibility or predisposition to a certain disease, then the detrimental nsSNV is also a deleterious one. Interestingly, the abundance of the detrimental mutations found in our work was comparable with but slightly higher than the previously reported estimated overall abundance of the moderately to strongly deleterious mutations in a single genome, which ranged between 29% and 49% [61,62]. 

We next studied the evolutionary conservation of genes harboring detrimental mutations. Specifically, we examined whether these genes tended to evolve at different rates compared to other well-characterized gene sets, such as cancer genes and housekeeping genes. To initiate this study, we defined “disruptive” genes as those carrying at least one deleterious nsSNV, as predicted by the SNP-IN tool. We then collected a cancer gene set from COSMIC (https://cancer.sanger.ac.uk/cosmic accessed on 10 August 2023) [63] and MutPanning [37], resulting in 987 genes with high confidence. Next, we considered a set of 3804 housekeeping genes (Figure 2d, see Section 2). After calculating the evolutionary rates (dN/dS) for each group (the dN/dS median values for housekeeping, detrimental, and cancer gene sets were 0.235 ± 0.158, 0.223 ± 0.159, and 0.197 ± 0.157, respectively), we found that the differences between the dN/dS ratios of the three gene sets were all significant (Figure 2e). Disruptive genes had a slightly higher dN/dS ratio compared to the cancer genes (*p* = 0.003). At the same time, disruptive genes were found to have a slightly lower ratio than the housekeeping genes (*p* = 0.004).

### 3.2. Genes Enriched with Detrimental Mutations Are Associated with Diverse Molecular Functions and Are Implicated in Various Diseases

Following the observation of widespread disruptions in the human interactome caused by common variants occurring in the healthy populations, we focused on the genes whose protein products are enriched with detrimental mutations, which is a more constrained set compared to the set of disruptive genes introduced earlier. Genes enriched with detrimental mutations were defined as those with their disruptive mutation rate larger than the average rate plus the standard deviation (see Section 2). Among all 3603 proteins that carried nsSNVs with the functional outcome annotated by the SNP-IN tool, there were 461 proteins (12.8%) enriched with detrimental mutations. 

To determine if the set of 461 genes enriched with detrimental mutations shared any functional patterns, we performed a GO enrichment analysis. We only selected the third-level GO terms in the GO hierarchy, which represented a reasonable trade-off between the level of detail each term could provide and how well that term was populated with the gene instances. In total, we extracted 458 GO terms with significant *p*-value after the multiple hypothesis correction (see Section 2). Examining the significant GO terms revealed a few interesting findings. First, in the molecular function category, we obtained 62 GO terms enriched in the gene set with significantly high number of detrimental nsSNVs. The majority of the top 20 significant molecular function GO terms (see Supplementary Figure S1) were related to molecular binding, including nucleotide binding (GO:0000166), TAP binding (GO:0046977), and pyridoxal phosphate binding (GO:0030170). This implied that while the detrimental mutations were expected to exclusively disrupt the protein–protein interactions, the genes carrying them could also be involved in other molecular interaction mechanisms. In the biological process GO term category, more than one-third of terms were related to the immune system response. The immune system consists of many molecular interactions and processes implemented to protect the organism against a wide variety of pathogens and diseases. In addition, the top two terms in the biological process category were small molecule and organic acid metabolic processes. Both kinds of these biological processes have been implicated in a number of common and rare genetic conditions, such as disorders in small molecules metabolism and organic acid disorders [64,65]. We note that besides the occurrence of a significantly high number of detrimental mutations on these genes, there was also an uneven distribution of these mutations across different populations. The differences in the resulting network rewiring effect could be linked to the population phenotypic variance, such as population-specific disease susceptibility. 

Generally speaking, such a disruptive effect on a protein–protein interaction is expected to alter or completely abolish the regular biological processes or molecular functions, potentially leading to a disease phenotype. Thus, we further investigated any potential links between these genes enriched with detrimental mutations and complex diseases. We examined the set of genes enriched with detrimental mutations using OMIM (https://www.omim.org/ accessed on 10 August 2023) [66] and HGMD (https://www.hgmd.cf.ac.uk/ac/index.php accessed on 10 August 2023) [67] databases. Indeed, while all these mutations originated from the genomes of healthy individuals, the 461 genes were associated with various complex diseases (see Supplementary Table S1). 

### 3.3. Edgotype Analysis Reveals Distinct Protein Edgetic Profiles in Major Populations

The human interactome, or the human protein–protein interaction network, is the set of protein–protein interactions that occur in a human cell. The interactome is represented as a graph, with nodes and edges corresponding to the individual proteins and their pairwise physical interactions, respectively. The edgetic network perturbation model, or “edgotype”, emphasizes the disruption of specific edges. However, unlike the traditional gene-centric model, which mainly focuses on the overactivity or silencing of gene expression, the edgotype can easily incorporate the influence of genetic variation. 

Utilizing the edgotype concept, we systematically characterized the edgetic profile for the genes and their proteins enriched with detrimental mutations across different populations. To simplify the analysis, we treated the mutations with the neutral and beneficial effects as the same group of mutations that preserve the protein–protein interaction, as opposed to the interaction-disrupting effect of the detrimental mutations. We then excluded the interaction-preserving mutations from constructing the edgetic profile. Thus, the edgetic profile of a protein in a healthy population was represented as a sequence of binary vectors. Given a protein–protein interaction that the protein of interest was involved in and the protein’s binary vector, 1 corresponded to a detrimental mutation with non-zero allele frequency in the population, and 0 corresponded to a detrimental mutation that was not present in this population. The difference between the gene edgetic profiles in two different populations is quantified using the Manhattan distance between the extended vectors (see Section 2). For each protein, we calculated the average pairwise edgetic profile difference between any two populations. This average difference was further normalized by the total number of detrimental mutations. Thus, the average difference value of 0.0 would correspond to a protein with an identical edgetic profile across all populations, indicating no differences in functional effects across the populations. On the other hand, the difference value of 1.0 would correspond to a protein with each detrimental mutation for each PPI present only in one population, indicating a unique combination of the interaction-disrupting mutations in each population. Our results showed that on average, the normalized difference of the total detrimental mutations occurring in one protein between any two major populations was 0.405. We also generated another 1000 random samples; Z-score and the corresponding *p*-values were calculated to assess the statistical significance. Our results show that the difference from the real 1000 Genomes Project dataset is significantly higher (*p*-value < 0.001; Supplementary Figure S5). Such a difference in edgetic profiles could be explained by the fact that a substantial number of mutations that were found to be detrimental to the PPI were also unique to a small subset of populations. Such a distinct prevalence of detrimental mutations in different populations, combined with the unique interactions these mutations’ targets, can help explain the genetic diversity and phenotypic variance across populations. 

### 3.4. Edgetic Properties of Population-Specific nsSNVs Could Help Explain the Phenotypic Variance across Different Populations 

**Figure 3:**
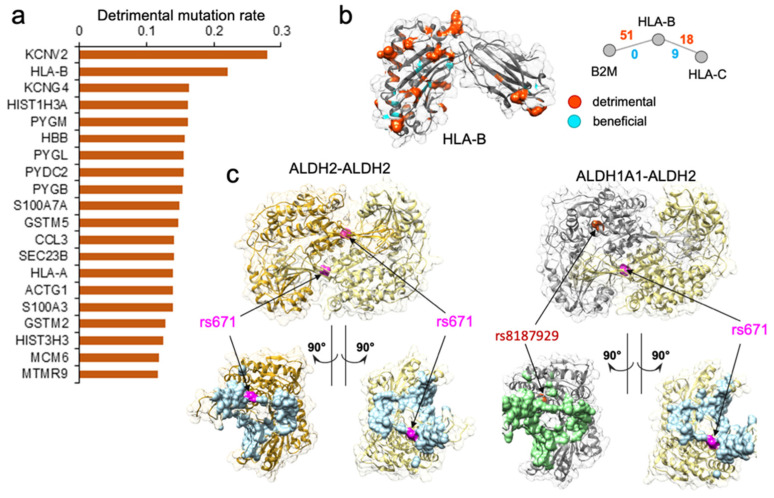
Genes enriched with detrimental mutations are associated with diverse molecular functions. (**a**) Top 20 genes with the highest disruptive mutation rate, normalized by protein sequence length. (**b**). Analysis of population-specific mutations affecting PPI mediated by HLA-B. HLA-B is one of the top genes with the highest rate of detrimental mutations regulating two PPIs, but also with a high number of beneficial nsSNVs. Shown is the protein structure of the HLA-B, with the residues corresponding to detrimental mutations highlighted in orange and beneficial mutations highlighted in cyan. Shown are the two PPIs mediated by HLA-B and the number of detrimental and beneficial mutations associated with each PPI. Detrimental mutations are widespread on the structure. (**c**) Case study of two protein–protein interactions implicated in the “Asian Flush” phenotype. Shown are two mutations, rs671 and rs8187929, mapped w.r.t protein binding interface colored in light blue for ALDH2 and light green for ALDH1A1. rs671 is predicted by the SNP-IN tool to disrupt the two PPIs. While rs8187929 is also related to human drinking behavior, it may be less effective than rs671.

Our computational predictions enabled the characterization of the edgetic property of genetic mutations. More importantly, these functional characterizations of mutations, together with the population-specific genotype information, enabled us to generate a concrete “molecular mechanism” hypothesis for certain complex phenotypes, and to explain the phenotypic variance across different populations. Our approach to analyzing the genotype–phenotype relationship could be well illustrated by an example of edgetic mutation rs671 on gene *ALDH2*. *ALDH2* gene encodes aldehyde dehydrogenase 2, a member of a family of enzymes that metabolizes alcohol, and plays a major role in ethanol catabolism [68]. Another gene, *ALDH1A1*, encodes retinal dehydrogenase 1, which oxidizes retinaldehyde [69], but also has a broader specificity and oxidizes other aldehydes [69]. The missense rs671 mutation of *ALDH2* gene was well known as the culprit in the phenomenon of “Asian Flush” [70,71], wherein Asian people develop a flush reaction, turning red in the face, neck, and shoulders, after drinking alcohol [72,73]. The frequency of individuals carrying rs671 was the highest in eastern Asia (MAF = 17.6%), but was almost absent among other major populations [72]. *ALDH2* was involved in two protein–protein interactions: one self-interaction which was the basis for its homo-oligomerization, and another one with *ALDH1A1*. According to the SNP-IN tool prediction, rs671 could disrupt both of these interactions (Figure 3c). *ALDH2* functioned as a homo-tetramer in the cell, and the rs671 allele resulted in a mutant ALDH2*2 protein which caused a critical disruption to ALDH2–ALDH2 protein-protein interaction. Such disruption destabilized the tetramer, interfered with catalytic activity, and increased the protein turnover [68]. As a result, the ALDH2*2 protein was made defective in metabolizing alcohol. It is worth noting that rs671 might also disrupt ALDH1A1–ALDH2 interaction, further reducing the ethanol catabolism. There was another mutation rs8187929 on gene *ALDH1A1* that could exert a similar impact on ALDH1A1–ALDH2 interaction (Figure 3c). A recent study reported that rs8187929 was related to alcohol consumption and drinking behaviors in the Japanese population [74]. Similar to rs671, rs8187929 also predominantly existed in the East Asian population, but with a much lower allele frequency (MAF = 4.56% in East Asians). Furthermore, while occurring in the same protein, the edgetic profile, which lists the interactions that were disrupted by the mutations, of rs671 was found to be clearly distinct from rs8187929. According to the edgetic profiling, rs8187929 had a more limited impact on the enzymatic activities involved in ethanol metabolism, potentially disrupting only one PPI, while rs671 had a substantially wider rewiring effect on the corresponding protein–protein interactions, with a higher prevalence in the East Asian population. 

We performed another in-depth study on *HLA-B* gene, which was among the genes with the highest disruptive mutation rate, 22.1% (Figure 3a,b). *HLA-B* (major histocompatibility complex, class I, B) is a human gene encoding a protein that plays an important role in the immune system, helping distinguish the organism’s native proteins from the foreign ones [75], and enabling the immune system to react to a broad spectrum of pathogens [76,77,78,79]. Together with two other related proteins, HLA-A and HLA-C, HLA-B protein belongs to the major histocompatibility complex (MHC) gene family. Interestingly, *HLA-A* was also found among the top 461 genes enriched with detrimental mutations (Supplementary Figure S2). The high number of normal variations disrupting protein–protein interactions suggested that diverse immune responses to various pathogens could be partially attributed to many distinct *HLA* gene edgetic profiles and the unique rewiring subnetwork centered around the HLA family. We hypothesize that in conjunction with the unequal distribution of mutation frequency across populations, the rewiring effect of the HLA complex could contribute to the different disease susceptibility and the varied immune response. 

### 3.5. Comparative Network Analysis Shows Disease Mutations Target Less Efficient Subnetworks 

**Figure 4:**
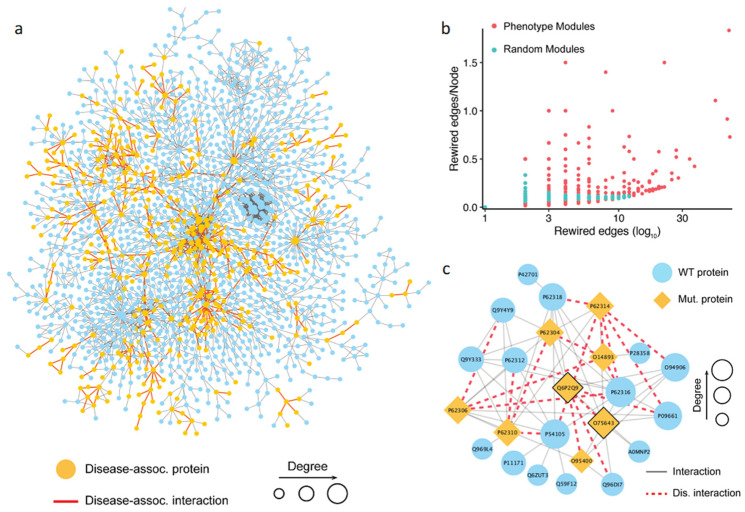
Analysis of phenotype-associated modules detected based on the topological properties. (**a**) Network visualization of the largest connected component targeted by the pathogenic mutations and common mutations. The yellow node represents a gene carrying pathogenic mutations, and the red edge represents an interaction targeted by both the pathogenic mutations and common mutations. (**b**) Comparison of interaction disruptions accumulated in the phenotype associated modules and random modules. The results show that a phenotype-associated module carries more detrimental mutations than a random one. (**c**) An example of the phenotype-associated module in the human interactome detected based on the DSD idea. Shown is a dense PPI module with mutant proteins carrying variations disrupting PPI interactions, indicated by the orange nodes for the proteins and red dashed edges for the PPIs, respectively.

We next compared the topological properties and the rewiring effects in the human interactome of both the pathogenic mutations curated from the ClinVar database and the population mutations from this study. First, we constructed the human interactome by utilizing two main protein–protein interaction data sources, HINT (High-quality INTeractomes, http://hint.yulab.org/ accessed on 10 August 2023) and HuRI (Human Reference Interactome, http://www.interactome-atlas.org/ accessed on 10 August 2023). The unified human interactome consisted of 105,087 interactions (see Methods). From the list of human disease genes retrieved from the ClinVar database, 576 genes carried at least one detrimental mutation, and the detrimental mutations occurring on these pathogenic genes rewire 1162 interactions. There were 3603 genes with at least one detrimental mutation from the 1000 Genomes Project, with the number of rewired interactions equal to 4529. The overlaps between the two gene sets and the two interaction sets were 449 genes and 736 interactions, respectively. The overlaps of genes and the affected interactions were relatively high, suggesting that both pathogenic mutations and normal variants could cause detrimental effects on the same subset of interactions (Figure 4a). We thus hypothesized that the presence of detrimental mutations in healthy populations might not be sufficient to cause the disease phenotype, but could nevertheless contribute to the disease susceptibility. 

We next considered two groups of disrupted interactions caused by nsSNVs in the context of the whole human interactome and examined their topological properties. To account for the exclusive effects elicited by each set of the mutations, we excluded the 736 interactions shared between the two datasets. Then, we measured the centrality of the interactions rewired by the two groups of mutations using two main network edge centrality measures, the shortest-path edge betweenness and the flow edge betweenness (see Section 2). Our results showed that there was no significant difference between the two disrupted interaction sets in terms of both edge centrality measures. Furthermore, the results showed that both the pathogenic mutations and the mutations found in healthy populations did not tend to disrupt interactions of high centrality, thus avoiding critical breakdowns in the interactome. We further investigated the network rewiring effect from a more indirect perspective, by collapsing the two disrupted interaction sets into unified subnetworks and examining their network efficiency, a measure that describes how efficiently a network exchanges information. Because protein–protein interaction networks have been previously determined to be a small-world network [80], the network efficiency is generally better preserved during the randomized attack, compared to an attack targeting the hub proteins [81]. We found that the subnetwork disrupted by the pathogenic mutations had a lower network efficiency compared to population-specific mutations, with a nearly two-fold difference (0.0117 vs. 0.0223). The fact that the pathogenic mutations target less efficient networks than the population mutations suggests that they occur, *en masse*, in more random positions of the interactome, while the occurrence of the population mutations appears to be more controlled and evolution-guided.

### 3.6. Phenotype-Associated Modules in the Human Interactome Are Enriched with Detrimental Mutations 

Modular structure is one of the essential characteristics of biological networks [17]. The identification of these modules is a key step in network analysis helping to uncover the biological mechanisms underlying complex phenotypes [20,82]. To identify biologically relevant modules in the human interactome, we applied two distinct module detection methods: the first method relied only on the network’s topological information, with no prior biological knowledge, while the second method was a seed-based approach that leveraged knowledge about the disease-associated proteins (see Section 2). Given its outstanding performance in the DREAM challenge [56], we expect the first method to accurately discover the modules that are significantly associated with complex traits, including both healthy phenotypes and disease phenotypes. For the second, biological knowledge-driven, approach, we adopted DIAMOnD [59]. The DIAMOnD method aimed to identify the full disease module around a set of known disease proteins (see Section 2).

Using the first approach, we collected 1055 possible phenotype-associated modules with high confidence, with the module sizes ranging between 3 and 100 genes. We further investigated whether the PPI disruptions and rewiring inside these phenotypes associated modules caused by detrimental mutations had any role in developing a complex trait. To do so, we randomly generated the same number of modules and compared the number of rewired edges between the two module sets. Then, the number of the rewired edges in the module was normalized by the total number of nodes in the module. The results showed that the phenotype-associated modules had significantly more detrimental mutations than the random ones (Figure 4b). This finding supported our hypothesis that the rewiring caused by the detrimental mutations inside the phenotype-associated modules may directly contribute to the development of certain phenotypes. Shown in Figure 4c is an example of detected module that includes 24 genes. The literature search confirmed that this module was associated with pre-mRNA splicing. Specifically, some genes in the module, such as PRPF8 (Uniprot: Q6P2Q9), GEMIN2 (Uniprot: O14893), and SNRNP200 (Uniprot: O75643), were the key components of the spliceosome, a complex molecular machine consisting of small nuclear RNAs (snRNAs) and approximately 80 proteins [83]. In this module, the detrimental mutations targeted interactions mediated by two protein hubs, PRPF8 and SNRNP200 (Figure 4). PRPF8 forms a scaffold to help in the assembly of the snRNAs and proteins of the complex, and SNRNP200 mediates the interaction of snRNAs and catalytic activity. Mutations in these proteins have been previously linked to a number of traits [84,85,86,87]. These traits include both normal phenotypes and disease phenotypes, such as leukocyte count, body mass index, balding measurement, coronary artery disease, and others. Although the phenotypic nature of the interaction-disrupting variants has not been determined, our results provide mechanistic insights into functional variation by pinpointing specific protein–protein interactions. 

**Figure 5:**
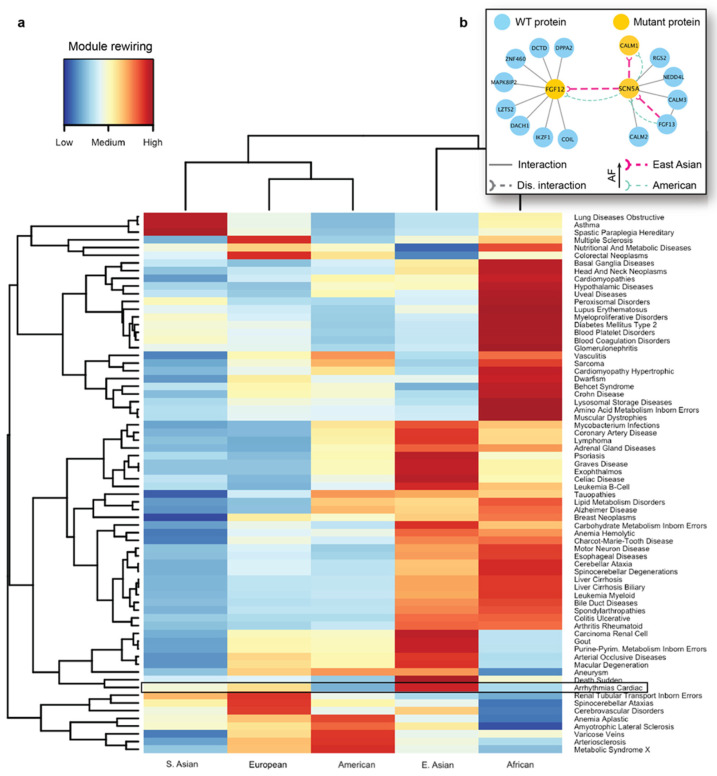
Analysis of disease modules impacted by detrimental mutations. (**a**) A heatmap showing the different prevalence and disruption levels caused by population-specific mutations across different populations. The disease-associated modules are represented by rows, and the populations are represented by columns. The color of each cell in the heatmap represents the prevalence of rewiring in a specific disease module for a given population. Cardiac arrhythmia module is highlighted to show the population-specific difference. (**b**) Key interactions damaged in the module associated with arrhythmias carry detrimental mutations with different prevalence in East Asians and Americans. *FGF12* and *SCN5A* are two important genes implicated in arrhythmias, and disruptions associated with *FGF12* and *SCN5A* are more frequent among East Asians compared to the American population.

We next carried out seed-based module detection on the human interactome. To do so, we first collected disease-associated genes as seeds for 70 complex diseases. We note again that these mutations originated from the healthy populations, hence the interaction disruptions caused by them were not expected to cause severe functional changes in the cell. However, we suspected that certain population-specific changes in these disease-associated modules caused by detrimental mutations could be linked to the differences in disease susceptibility across different ethnic groups. We then ran the DIAMOnD algorithm with the disease-associated genes as partial input. For the 70 disease modules collected from DIAMOnD output, we summarized the network-aggregated results in the form of a heatmap (Figure 5a). In the heatmap, the disease-associated modules and the populations were represented by rows and columns, respectively. The color of each cell in the heatmap represented the prevalence of rewiring in a specific disease module and a population, where red represented a higher occurrence of disrupted interactions in the module. To uncover patterns, we also applied hierarchical clustering to group diseases and populations by the similarity of rewiring prevalence. As a result, we found that among the five major populations, detrimental mutations and the resulting PPI rewiring were most prevalent in East Asians and Africans across 70 disease modules.

As an in-depth study, we investigated the disease module for cardiac arrhythmia (Supplementary Figure S3). A cardiac arrhythmia is a group of conditions that cause the heart to beat too fast, too slow, or irregularly in timing [88]. Arrhythmia affects millions of people and usually develops in older adults, with many affected people not requiring medical attention or treatment. However, this condition can also have lethal outcomes, such as sudden cardiac death. Even though there are far fewer cardiac genetic studies in Asian populations than in Western populations, a recent review on arrhythmias [89] found that Japanese individuals carry a higher prevalence of long QT syndrome and Brugada syndrome, which can both increase the risk of sudden cardiac death. Several well-characterized proteins associated with arrhythmias are known to interact with each other. A protein–protein interaction associated with Brugada syndrome and sudden death is formed between FGF12 (Fibroblast growth factor 12) and SCN5A (sodium voltage-gated channel alpha subunit 5) proteins. Fibroblast growth factor homologs (FGF11–FGF14) perform many intracellular functions and are well known to bind to sodium and calcium channels, modulating cardiac currents. Among them, FGF12 is the major fibroblast growth factor expressed in the human cardiac ventricle. Another key protein, SCN5A is a sodium channel that plays an important role in modulating electrical impulses and their conduction in the heart. SCN5A mutations [90] are implicated in many arrhythmias such as long QT syndrome, Brugada syndrome, atrial fibrillation, progressive cardiac conduction defect, and sick sinus syndrome. In addition, SCN5A is known to interact with the calmodulin gene products (CALM1, CALM2, and CALM3) that are also associated with susceptibility to arrhythmias [91].

As indicated by the heatmap, there was a substantial difference in the overall PPI rewiring prevalence between East Asian and American populations (Figure 5a). When we selected the disrupted interactions of each of these populations and mapped them to the arrhythmia-associated module (Supplementary Figure S3), we saw distinct rewiring patterns in the PPIs associated with arrhythmias in East Asians and Americans. We further focused on a few specific interactions mediated by the aforementioned arrhythmia-associated proteins (Figure 5b). For the interaction between FGF12 and SCN5A, we predicted rewiring due to mutations in FGF12, in which the deleterious variant is more frequent in East Asians than in Americans (Figure 5b, thick and thin edges, respectively). This PPI disruption by the mutant proteins had been experimentally studied in mice and humans [92]; it has been demonstrated that such disruption can cause a sodium channel loss-of-function phenotype. More specifically, experiments revealed the reduced binding of the mutant FGF12 to SCN5A and the resulting reduction in sodium channel current, which affected the cardiac ventricular action potential. Next, our analysis showed that the disruption (Figure 5b) between SCN5A and CALM1 was also more frequent in the East Asian population. Finally, we predicted another interaction of SCN5A, with FGF13, to be disrupted, due to a detrimental variant in the former protein. The SCN5A-FGF13 PPI was also implicated in arrhythmias [93]. This analysis provided us with the mechanistic explanation of the molecular mechanisms driving the increased susceptibility of the disease in the selected populations.

## 4. Discussion

In this work, we created a comprehensive catalog of population-specific edgetic effects at the whole-interactome level. Our work leveraged a recently developed machine learning approach, the SNP-IN tool, that determined the interaction-rewiring effects of nsSNVs. This method was previously applied to variants associated with diseases, showing the feasibility of a large-scale in silico edgetic study. To compare the functional impact of the population-specific variations, we applied our approach to annotate the protein-rewiring effects of a comprehensive set of 46,599 nsSNVs collected from the 1000 Genomes Project. The functional impact of a variant on a PPI was annotated neutral, detrimental, or beneficial based on their effect on the PPI, quantified by a change in the binding affinity. In summary, we determined that 25,185 SNVs (54%) were predicted detrimental to at least one PPI. PPIs often mediate molecular functions for the interacting proteins, thus one can expect that many (albeit not necessarily all) mutations that disrupt a PPI will disrupt a certain molecular function mediated by this PPI. Changes in the molecular function are, in turn, often, but not always, associated with the changes in phenotype. Further experiments are needed to ascertain which PPI-rewiring mutations directly influence the phenotype, which would offer a clear mechanistic insight into the observed phenotypic diversity.

Our findings on the dN/dS ratios of the disruptive gene set showed a slight difference between the ratios of the disruptive gene set and those of the two other sets, the housekeeping genes and cancer drivers, both of which are known to be evolutionarily conservative. The differences are statistically significant, suggesting that the disruption of protein–protein interaction caused by nsSNVs imposes unique functional constraints on the human genome’s evolution. We also note that the differences are small, so one should use caution when making conclusions from the analysis.

While the traditional “node-centric” gene removal approach has been widely used to quantify the disruption in the network due to the gene loss, the edgotype approach provides a much finer-grain view of the mutation’s functional impact by focusing on alterations in specific (not necessarily all) molecular interactions mediated by the mutated protein. Indeed, many mutations have been shown to be edgetic [24,31]. Besides, the interactions mediated by the same protein may not necessarily occur at the same time. Thus, the mutations accumulating on the same protein might affect different PPIs at different times. Edgetic network perturbation models have also been proposed and adopted to study complex genetic diseases [22,24,26,31,94]. Sahni et al. suggested a relationship between the edgetic perturbations and disease severity [24]. The edgotype provided a plausible explanation for some complicated genetic phenomena, such as locus heterogeneity and gene pleiotropy [26,95]. In addition, the edgetic perturbation model offered a network-based hypothesis to explain modes of inheritance [22]. While all these studies have been focused on the disease-associated mutations, the current work suggests that the edgotype concept can be extended to population genetics to study the mutation frequency patterns and their functional impacts across healthy populations. Case studies in this work provide evidence that this edgotype-based approach can better explain the phenotypic variance across populations. 

Our analysis revealed that genes enriched with detrimental mutations were linked to various disease phenotypes. Because these mutations were harbored among the healthy individuals, the mutations’ interaction-disrupting effects alone would not be enough to result in a disease phenotype. Instead, a distinct group of mutations, absent in the healthy population but present in the same genes, might inflict more severe damage on the molecular mechanisms connected with the disease. Nonetheless, the detrimental mutations in the healthy population could add to the cumulative damage to protein–protein interaction, thereby potentially increasing susceptibility to the disease. This hypothesis received further supporting evidence from our comparative network analysis concerning disease mutations and normal mutations. This analysis shows that some of the healthy population mutations can target the same set of interactions that were previously shown to be targeted by the pathogenic mutations, leading to similar alterations in the network topology.

Finally, our research reveals noteworthy implications of detrimental mutations and their effects on network rewiring. We observed distinct population-specific rewiring patterns within disease modules, pointing to inherent differences in disease susceptibility across populations. Delving deeper into the network disease modules, our analysis uncovered that modules associated with specific phenotypes had a higher concentration of detrimental mutations compared to random modules. Moreover, when examining the effects of these mutations across different populations, the rewiring patterns within the disease-related network modules frequently emerged as unique to one or several populations. Overall, our large-scale in silico approach sheds light on the mechanistic nuances of population-specific differences, enhancing our understanding of both health and disease traits.

## Data Availability

Data is contained within Supplementary Material.

## Supplementary Materials

The following supporting information can be downloaded at: www.mdpi.com/xxx/s1. Figure S1: Top 20 enriched GO terms in three basic GO categories: biological process, molecular function and cellular component; Figure S2: HLA-A is one of genes carrying high number of normal disruptive mutation in the HLA gene family; Figure S3: Distinct rewiring patterns in interactome modules associated with arrhythmias in East Asians and Americans; Figure S4: Illustration of the concept of Diffusion State Distance (DSD); Figure S5: The reported normalized difference of the total detrimental mutations occurring in one protein between any two major populations is statistically significantly larger compared to the population of randomly shuffled edgetic profiles; Table S1: Disease phenotype information with disruptive mutation curated from OMIM, HGMD associated with genes enriched.

## References

[1] Schuster S.C. (2008). Next-generation sequencing transforms today’s biology. Nat. Methods.

[2] Metzker M.L. (2010). Sequencing technologies—The next generation. Nat. Rev. Genet..

[3] Shendure J., Balasubramanian S., Church G.M., Gilbert W., Rogers J., Schloss J.A., Waterston R.H. (2017). DNA sequencing at 40: Past, present and future. Nature.

[4] Consortium I.H. (2003). The international HapMap project. Nature.

[5] Consortium G.P. (2015). A global reference for human genetic variation. Nature.

[6] van Rooij J.G., Jhamai M., Arp P.P., Nouwens S.C., Verkerk M., Hofman A., Ikram M.A., Verkerk A.J., van Meurs J.B., Rivadeneira F. (2017). Population-specific genetic variation in large sequencing data sets: Why more data is still better. Eur. J. Hum. Genet..

[7] Consortium G.P. (2012). An integrated map of genetic variation from 1092 human genomes. Nature.

[8] Sherry S.T., Ward M.-H., Kholodov M., Baker J., Phan L., Smigielski E.M., Sirotkin K. (2001). dbSNP: The NCBI database of genetic variation. Nucleic Acids Res..

[9] Alexander R.P., Fang G., Rozowsky J., Snyder M., Gerstein M.B. (2010). Annotating non-coding regions of the genome. Nat. Rev. Genet..

[10] Cui H., Dhroso A., Johnson N., Korkin D. (2015). The variation game: Cracking complex genetic disorders with NGS and omics data. Methods.

[11] Pabinger S., Dander A., Fischer M., Snajder R., Sperk M., Efremova M., Krabichler B., Speicher M.R., Zschocke J., Trajanoski Z. (2014). A survey of tools for variant analysis of next-generation genome sequencing data. Brief. Bioinform..

[12] Cooper G.M., Shendure J. (2011). Needles in stacks of needles: Finding disease-causal variants in a wealth of genomic data. Nat. Rev. Genet..

[13] Ward L.D., Kellis M. (2012). Interpreting noncoding genetic variation in complex traits and human disease. Nat. Biotechnol..

[14] Raphael B.J., Dobson J.R., Oesper L., Vandin F. (2014). Identifying driver mutations in sequenced cancer genomes: Computational approaches to enable precision medicine. Genome Med..

[15] Cline M.S., Karchin R. (2011). Using bioinformatics to predict the functional impact of SNVs. Bioinformatics.

[16] Zhao N., Han J.G., Shyu C.-R., Korkin D. (2014). Determining effects of non-synonymous SNPs on protein-Protein interactions using supervised and semi-supervised Learning. PLoS Comput. Biol..

[17] Barabasi A.-L., Oltvai Z.N. (2004). Network biology: Understanding the cell’s functional organization. Nat. Rev. Genet..

[18] Zhang B., Tian Y., Zhang Z. (2014). Network biology in medicine and beyond. Circ. Cardiovasc. Genet..

[19] Carter H., Hofree M., Ideker T. (2013). Genotype to phenotype via network analysis. Curr. Opin. Genet. Dev..

[20] Ideker T., Sharan R. (2008). Protein networks in disease. Genome Res..

[21] Sahni N., Yi S., Zhong Q., Jailkhani N., Charloteaux B., Cusick M.E., Vidal M. (2013). Edgotype: A fundamental link between genotype and phenotype. Curr. Opin. Genet. Dev..

[22] Zhong Q., Simonis N., Li Q.R., Charloteaux B., Heuze F., Klitgord N., Tam S., Yu H., Venkatesan K., Mou D. (2009). Edgetic perturbation models of human inherited disorders. Mol. Syst. Biol..

[23] Dreze M., Charloteaux B., Milstein S., Vidalain P.-O., Yildirim M.A., Zhong Q., Svrzikapa N., Romero V., Laloux G., Brasseur R. (2009). ‘Edgetic’perturbation of a C. elegans BCL2 ortholog. Nat. Methods.

[24] Sahni N., Yi S., Taipale M., Bass J.I.F., Coulombe-Huntington J., Yang F., Peng J., Weile J., Karras G.I., Wang Y. (2015). Widespread macromolecular interaction perturbations in human genetic disorders. Cell.

[25] Madhani H.D., Styles C.A., Fink G.R. (1997). MAP kinases with distinct inhibitory functions impart signaling specificity during yeast differentiation. Cell.

[26] Wang X., Wei X., Thijssen B., Das J., Lipkin S.M., Yu H. (2012). Three-dimensional reconstruction of protein networks provides insight into human genetic disease. Nat. Biotechnol..

[27] Vidal M., Cusick M.E., Barabási A.-L. (2011). Interactome networks and human disease. Cell.

[28] Das J., Yu H. (2012). HINT: High-quality protein interactomes and their applications in understanding human disease. BMC Syst. Biol..

[29] Luck K., Kim D.K., Lambourne L., Spirohn K., Begg B.E., Bian W., Brignall R., Cafarelli T., Campos-Laborie F.J., Charloteaux B. (2020). A reference map of the human protein interactome. Nature.

[30] Wang K., Li M., Hakonarson H. (2010). ANNOVAR: Functional annotation of genetic variants from high-throughput sequencing data. Nucleic Acids Res..

[31] Cui H., Zhao N., Korkin D. (2018). Multilayer View of Pathogenic SNVs in Human Interactome through In Silico Edgetic Profiling. J. Mol. Biol..

[32] Benedix A., Becker C.M., de Groot B.L., Caflisch A., Böckmann R.A. (2009). Predicting free energy changes using structural ensembles. Nat. Methods.

[33] Moal I.H., Fernández-Recio J. (2012). SKEMPI: A Structural Kinetic and Energetic database of Mutant Protein Interactions and its use in empirical models. Bioinformatics.

[34] Sussman J.L., Lin D., Jiang J., Manning N.O., Prilusky J., Ritter O., Abola E.E. (1998). Protein Data Bank (PDB): Database of three-dimensional structural information of biological macromolecules. Acta Crystallogr. D Biol. Crystallogr..

[35] Fiser A., Šali A. (2003). Modeller: Generation and refinement of homology-based protein structure models. Methods in Enzymology.

[36] Sondka Z., Bamford S., Cole C.G., Ward S.A., Dunham I., Forbes S.A. (2018). The COSMIC Cancer Gene Census: Describing genetic dysfunction across all human cancers. Nat. Rev. Cancer.

[37] Dietlein F., Weghorn D., Taylor-Weiner A., Richters A., Reardon B., Liu D., Lander E.S., Van Allen E.M., Sunyaev S.R. (2020). Identification of cancer driver genes based on nucleotide context. Nat. Genet..

[38] Eisenberg E., Levanon E.Y. (2013). Human housekeeping genes, revisited. Trends Genet..

[39] Zhu J., He F., Hu S., Yu J. (2008). On the nature of human housekeeping genes. Trends Genet..

[40] Butte A.J., Dzau V.J., Glueck S.B. (2001). Further defining housekeeping, or “maintenance,” genes Focus on “A compendium of gene expression in normal human tissues”. Physiol. Genom..

[41] Kimura M. (1968). Evolutionary rate at the molecular level. Nature.

[42] Kinsella R.J., Kähäri A., Haider S., Zamora J., Proctor G., Spudich G., Almeida-King J., Staines D., Derwent P., Kerhornou A. (2011). Ensembl BioMarts: A hub for data retrieval across taxonomic space. Database.

[43] (2017). UniProt: The universal protein knowledgebase. Nucleic Acids Res..

[44] Ashburner M., Ball C.A., Blake J.A., Botstein D., Butler H., Cherry J.M., Davis A.P., Dolinski K., Dwight S.S., Eppig J.T. (2000). Gene Ontology: Tool for the unification of biology. Nat. Genet..

[45] Huang D.W., Sherman B.T., Lempicki R.A. (2009). Bioinformatics enrichment tools: Paths toward the comprehensive functional analysis of large gene lists. Nucleic Acids Res..

[46] Huang D.W., Sherman B.T., Lempicki R.A. (2009). Systematic and integrative analysis of large gene lists using DAVID bioinformatics resources. Nat. Protoc..

[47] Freeman L.C. (1977). A set of measures of centrality based on betweenness. Sociometry.

[48] Koschützki D., Schreiber F. (2008). Centrality analysis methods for biological networks and their application to gene regulatory networks. Gene Regul. Syst. Biol..

[49] Mizruchi M.S., Mariolis P., Schwartz M., Mintz B. (1986). Techniques for disaggregating centrality scores in social networks. Sociol. Methodol..

[50] Puzis R., Altshuler Y., Elovici Y., Bekhor S., Shiftan Y., Pentland A. (2013). Augmented betweenness centrality for environmentally aware traffic monitoring in transportation networks. J. Intell. Transp. Syst..

[51] Girvan M., Newman M.E. (2002). Community structure in social and biological networks. Proc. Natl. Acad. Sci. USA.

[52] Newman M.E. (2005). A measure of betweenness centrality based on random walks. Soc. Netw..

[53] Tripathi S., Moutari S., Dehmer M., Emmert-Streib F. (2016). Comparison of module detection algorithms in protein networks and investigation of the biological meaning of predicted modules. BMC Bioinform..

[54] Vlaic S., Conrad T., Tokarski-Schnelle C., Gustafsson M., Dahmen U., Guthke R., Schuster S. (2018). ModuleDiscoverer: Identification of regulatory modules in protein-protein interaction networks. Sci. Rep..

[55] Cao M., Zhang H., Park J., Daniels N.M., Crovella M.E., Cowen L.J., Hescott B. (2013). Going the distance for protein function prediction: A new distance metric for protein interaction networks. PLoS ONE.

[56] Choobdar S., Ahsen M.E., Crawford J., Tomasoni M., Fang T., Lamparter D., Lin J., Hescott B., Hu X., Mercer J. (2015). Assessment of network module identification across complex diseases. PLoS Comput. Biol..

[57] Cao M., Pietras C.M., Feng X., Doroschak K.J., Schaffner T., Park J., Zhang H., Cowen L.J., Hescott B.J. (2014). New directions for diffusion-based network prediction of protein function: Incorporating pathways with confidence. Bioinformatics.

[58] Pedregosa F., Varoquaux G., Gramfort A., Michel V., Thirion B., Grisel O., Blondel M., Prettenhofer P., Weiss R., Dubourg V. (2011). Scikit-learn: Machine learning in Python. J. Mach. Learn. Res..

[59] Ghiassian S.D., Menche J., Barabási A.-L. (2015). A DIseAse MOdule Detection (DIAMOnD) algorithm derived from a systematic analysis of connectivity patterns of disease proteins in the human interactome. PLoS Comput. Biol..

[60] Landrum M.J., Chitipiralla S., Brown G.R., Chen C., Gu B., Hart J., Hoffman D., Jang W., Kaur K., Liu C. (2020). ClinVar: Improvements to accessing data. Nucleic Acids Res..

[61] Subramanian S. (2012). The abundance of deleterious polymorphisms in humans. Genetics.

[62] Boyko A.R., Williamson S.H., Indap A.R., Degenhardt J.D., Hernandez R.D., Lohmueller K.E., Adams M.D., Schmidt S., Sninsky J.J., Sunyaev S.R. (2008). Assessing the evolutionary impact of amino acid mutations in the human genome. PLoS Genet..

[63] Tate J.G., Bamford S., Jubb H.C., Sondka Z., Beare D.M., Bindal N., Boutselakis H., Cole C.G., Creatore C., Dawson E. (2019). COSMIC: The catalogue of somatic mutations in cancer. Nucleic Acids Res..

[64] Guerrero R.B., Salazar D., Tanpaiboon P. (2018). Laboratory diagnostic approaches in metabolic disorders. Ann. Transl. Med..

[65] Ramsay J., Morton J., Norris M., Kanungo S. (2018). Organic acid disorders. Ann. Transl. Med..

[66] Hamosh A., Scott A.F., Amberger J.S., Bocchini C.A., McKusick V.A. (2005). Online Mendelian Inheritance in Man (OMIM), a knowledgebase of human genes and genetic disorders. Nucleic Acids Res..

[67] Stenson P.D., Ball E.V., Mort M., Phillips A.D., Shiel J.A., Thomas N.S., Abeysinghe S., Krawczak M., Cooper D.N. (2003). Human gene mutation database (HGMD®): 2003 update. Hum. Mutat..

[68] Macgregor S., Lind P.A., Bucholz K.K., Hansell N.K., Madden P.A., Richter M.M., Montgomery G.W., Martin N.G., Heath A.C., Whitfield J.B. (2009). Associations of ADH and ALDH2 gene variation with self report alcohol reactions, consumption and dependence: An integrated analysis. Hum. Mol. Genet..

[69] Agarwal D.P., Goedde H.W. (1989). Human aldehyde dehydrogenases: Their role in alcoholism. Alcohol.

[70] Wall T.L., Horn S.M., Johnson M.L., Smith T.L., Carr L.G. (2000). Hangover symptoms in Asian Americans with variations in the aldehyde dehydrogenase (ALDH2) gene. J. Stud. Alcohol.

[71] Cook T.A., Luczak S.E., Shea S.H., Ehlers C.L., Carr L.G., Wall T.L. (2005). Associations of ALDH2 and ADH1B genotypes with response to alcohol in Asian Americans. J. Stud. Alcohol.

[72] Eng M.Y., Luczak S.E., Wall T.L. (2007). ALDH2, ADH1B, and ADH1C genotypes in Asians: A literature review. Alcohol Res. Health.

[73] Ye L. (2009). Alcohol and the Asian flush reaction. SURG J..

[74] Matoba N., Akiyama M., Ishigaki K., Kanai M., Takahashi A., Momozawa Y., Ikegawa S., Ikeda M., Iwata N., Hirata M. (2020). GWAS of 165,084 Japanese individuals identified nine loci associated with dietary habits. Nat. Hum. Behav..

[75] Shankarkumar U. (2004). The human leukocyte antigen (HLA) system. Int. J. Hum. Genet..

[76] Hildebrand W.H., Domena J.D., Shen S.Y., Lau M., Terasaki P.I., Bunce M., Marsh S.G., Guttridge M.G., Bias W.B., Parham P. (1994). HLA-B15: A widespread and diverse family of HLA-B alleles. Tissue Antigens.

[77] Bihl F., Frahm N., Di Giammarino L., Sidney J., John M., Yusim K., Woodberry T., Sango K., Hewitt H.S., Henry L. (2006). Impact of HLA-B alleles, epitope binding affinity, functional avidity, and viral coinfection on the immunodominance of virus-specific CTL responses. J. Immunol..

[78] Williams F., Meenagh A., Darke C., Acosta A., Daar A., Gorodezky C., Hammond M., Nascimento E., Middleton D. (2001). Analysis of the distribution of HLA-B alleles in populations from five continents. Hum. Immunol..

[79] Khan M.A. (1995). HLA-B27 and its subtypes in world populations. Curr. Opin. Rheumatol..

[80] Goldberg D.S., Roth F.P. (2003). Assessing experimentally derived interactions in a small world. Proc. Natl. Acad. Sci. USA.

[81] Lo C.-Y.Z., Su T.-W., Huang C.-C., Hung C.-C., Chen W.-L., Lan T.-H., Lin C.-P., Bullmore E.T. (2015). Randomization and resilience of brain functional networks as systems-level endophenotypes of schizophrenia. Proc. Natl. Acad. Sci. USA.

[82] Barabási A.-L., Gulbahce N., Loscalzo J. (2011). Network medicine: A network-based approach to human disease. Nat. Rev. Genet..

[83] Will C.L., Lührmann R. (2011). Spliceosome structure and function. Cold Spring Harb. Perspect. Biol..

[84] Arzalluz-Luque Á., Cabrera J.L., Skottman H., Benguria A., Bolinches-Amorós A., Cuenca N., Lupo V., Dopazo A., Tarazona S., Delás B. (2021). Mutant PRPF8 causes widespread splicing changes in spliceosome components in retinitis pigmentosa patient iPSC-derived RPE cells. Front. Neurosci..

[85] Kurtovic-Kozaric A., Przychodzen B., Singh J., Konarska M.M., Clemente M.J., Otrock Z.K., Nakashima M., Hsi E.D., Yoshida K., Shiraishi Y. (2015). PRPF8 defects cause missplicing in myeloid malignancies. Leukemia.

[86] Zhang T., Bai J., Zhang X., Zheng X., Lu N., Liang Z., Lin L., Chen Y. (2021). SNRNP200 mutations cause autosomal dominant retinitis pigmentosa. Front. Med..

[87] Zhang X., Lai T.Y., Chiang S.W., Tam P.O., Liu D.T., Chan C.K., Pang C., Zhao C., Chen L. (2013). Contribution of SNRNP200 sequence variations to retinitis pigmentosa. Eye.

[88] Kong M.H., Fonarow G.C., Peterson E.D., Curtis A.B., Hernandez A.F., Sanders G.D., Thomas K.L., Hayes D.L., Al-Khatib S.M. (2011). Systematic review of the incidence of sudden cardiac death in the United States. J. Am. Coll. Cardiol..

[89] Offerhaus J.A., Bezzina C.R., Wilde A.A. (2019). Epidemiology of inherited arrhythmias. Nat. Rev. Cardiol..

[90] Ruan Y., Liu N., Priori S.G. (2009). Sodium channel mutations and arrhythmias. Nat. Rev. Cardiol..

[91] Makita N., Yagihara N., Crotti L., Johnson C.N., Beckmann B.-M., Roh M.S., Shigemizu D., Lichtner P., Ishikawa T., Aiba T. (2014). Novel calmodulin mutations associated with congenital arrhythmia susceptibility. Circ. Cardiovasc. Genet..

[92] Hennessey J.A., Marcou C.A., Wang C., Wei E.Q., Wang C., Tester D.J., Torchio M., Dagradi F., Crotti L., Schwartz P.J. (2013). FGF12 is a candidate Brugada syndrome locus. Heart Rhythm.

[93] Musa H., Kline C.F., Sturm A.C., Murphy N., Adelman S., Wang C., Yan H., Johnson B.L., Csepe T.A., Kilic A. (2015). SCN5A variant that blocks fibroblast growth factor homologous factor regulation causes human arrhythmia. Proc. Natl. Acad. Sci. USA.

[94] Kataka E., Zaucha J., Frishman G., Ruepp A., Frishman D. (2020). Edgetic perturbation signatures represent known and novel cancer biomarkers. Sci. Rep..

[95] Mosca R., Tenorio-Laranga J., Olivella R., Alcalde V., Céol A., Soler-Lopez M., Aloy P. (2015). dSysMap: Exploring the edgetic role of disease mutations. Nat. Methods.

