# Supplementary material for: The Extent of Edgetic Perturbations in the Human Interactome Caused by Population-Specific Mutations
Source: Biomolecules. 2023 Dec 27;14(1):0. doi: 10.3390/biom14010040 (PMC11154503; doi:10.3390/biom14010040)
Supplement: Supplementary file 1 [file biomolecules-14-00040-s001.zip › biomolecules-2580918-supplementary_final.pdf]

The Extent of Edgetic Perturbations in the Human Interactome Caused by Population-Specific Mutations

Hongzhu Cui [1,2,*], Suhas Srinivasan [3,4,5], Ziyang Gao [1] and Dmitry Korkin [1,3,6,*]

1 Bioinformatics and Computational Biology Program, Worcester Polytechnic Institute, Worcester, MA 01609, USA

2 Chromatography and Mass Spectrometry Division, Thermo Fisher Scientific, San Jose, CA 95134, USA

3 Data Science Program, Worcester Polytechnic Institute, Worcester, MA 01609, USA

4 Program in Epithelial Biology, Stanford School of Medicine, Stanford, CA 94305, USA

5 Center for Personal Dynamic Regulomes, Stanford School of Medicine, Stanford, CA 94305, USA

6 Computer Science Department, Worcester Polytechnic Institute, Worcester, MA 01609, USA

* Correspondence: cuihzh@gmail.com (H.C.); korkin@korkinlab.org (D.K.)

Supplementary Information

List of figures:

**Supplementary Figure S1**	Top 20 enriched GO terms in three basic GO categories: biological process, molecular function and cellular component.
**Supplementary Figure S2**	HLA-A is one of genes carrying high number of normal disruptive mutation in the HLA gene family.
**Supplementary Figure S3**	Supplementary Figure S3. Distinct rewiring patterns in interactome modules associated with arrhythmias in East Asians and Americans.
**Supplementary Figure S4**	Illustration of the concept of Diffusion State Distance (DSD).
**Supplementary Figure S5**	The reported normalized difference of the total detrimental mutations occurring in one protein between any two major populations is statistically significantly larger compared to the population of randomly shuffled edgetic profiles.

List of tables:

**Supplementary Table S1**	Disease phenotype information associated with genes enriched with disruptive mutation curated from OMIM, HGMD.

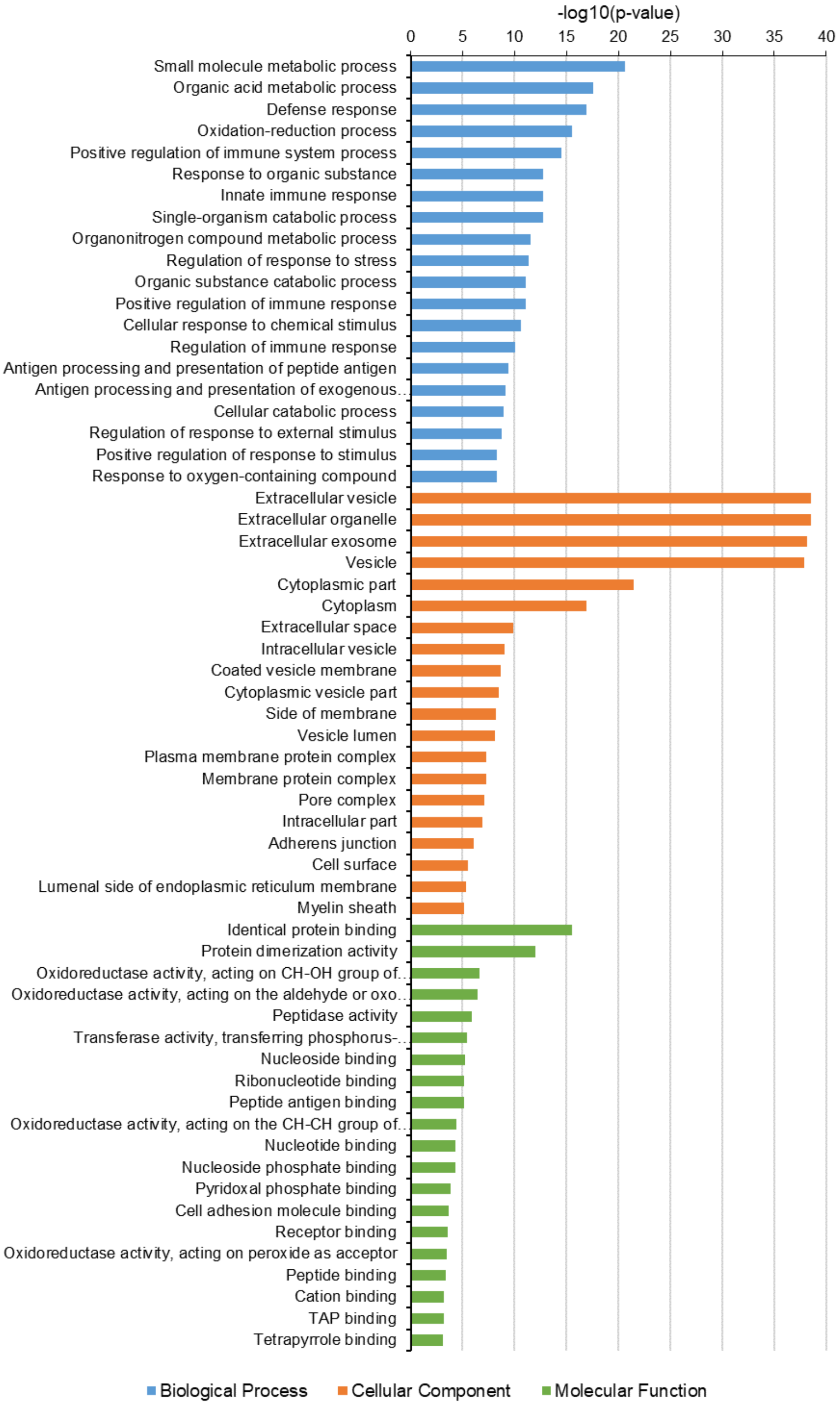

Supplementary Figure S1. Top 20 significant GO terms for genes enriched with disruptive mutations among three basic GO categories: biological process, molecular function and cellular component.

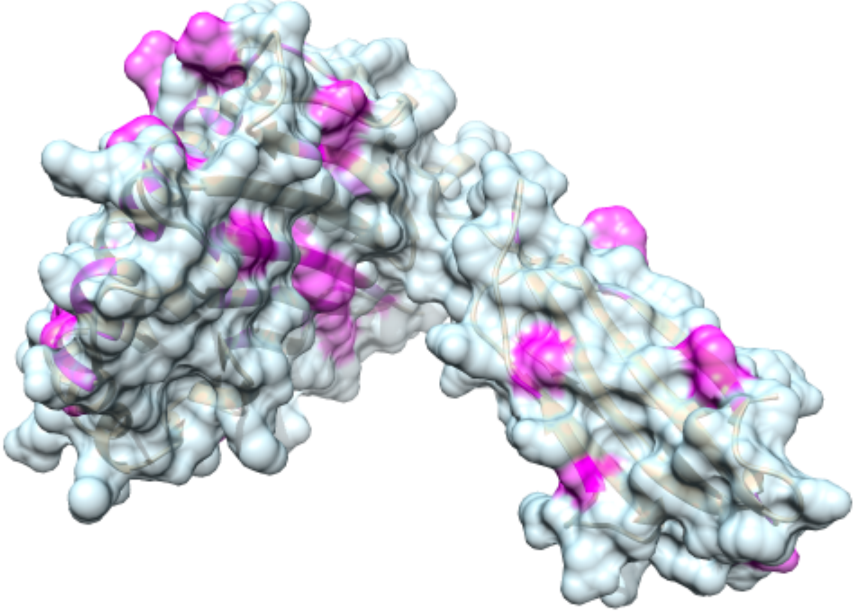

Supplementary Figure S2. HLA-A is one of the top genes carrying high number of normal disruptive mutation in the HLA gene family.

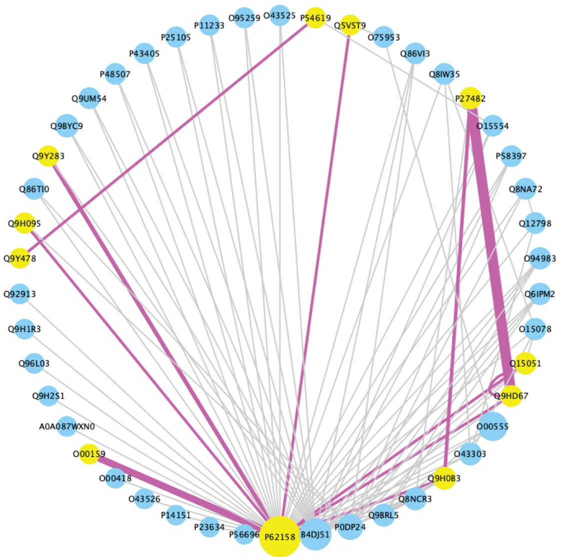

East Asian

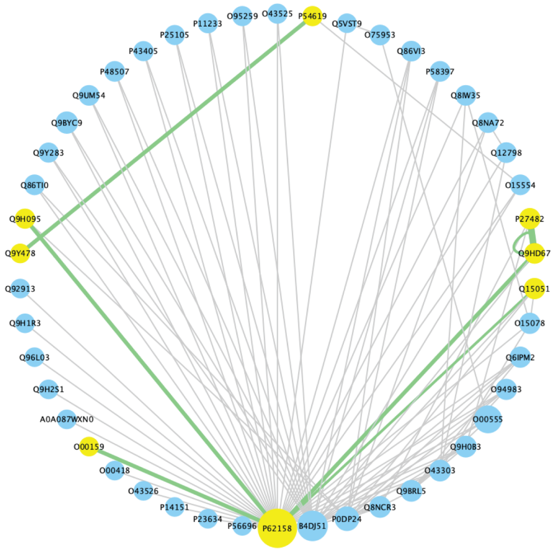

American

Supplementary Figure S3. Distinct rewiring patterns in interactome modules associated with arrhythmias in East Asians and Americans. The East Asian population has 11 proteins that carry mutations (yellow nodes) resulting in 11 rewired interactions (magenta edges), whereas the American population has 6 mutant proteins (yellow nodes) with 6 rewired interactions (green edges). Additionally, the higher allele frequency of a mutation and the corresponding rewired interaction is represented by the increasing thickness of edges, where one can see that the East Asian population has higher frequency of these mutations.

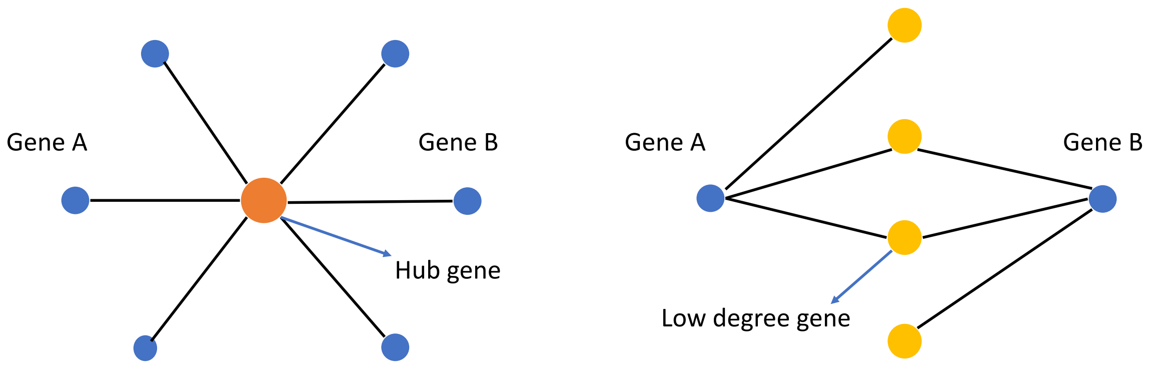

Supplementary Figure S4. Illustration of the concept of Diffusion State Distance (DSD). DSD downweighs the influence of the hub gene and favors low degree gene.

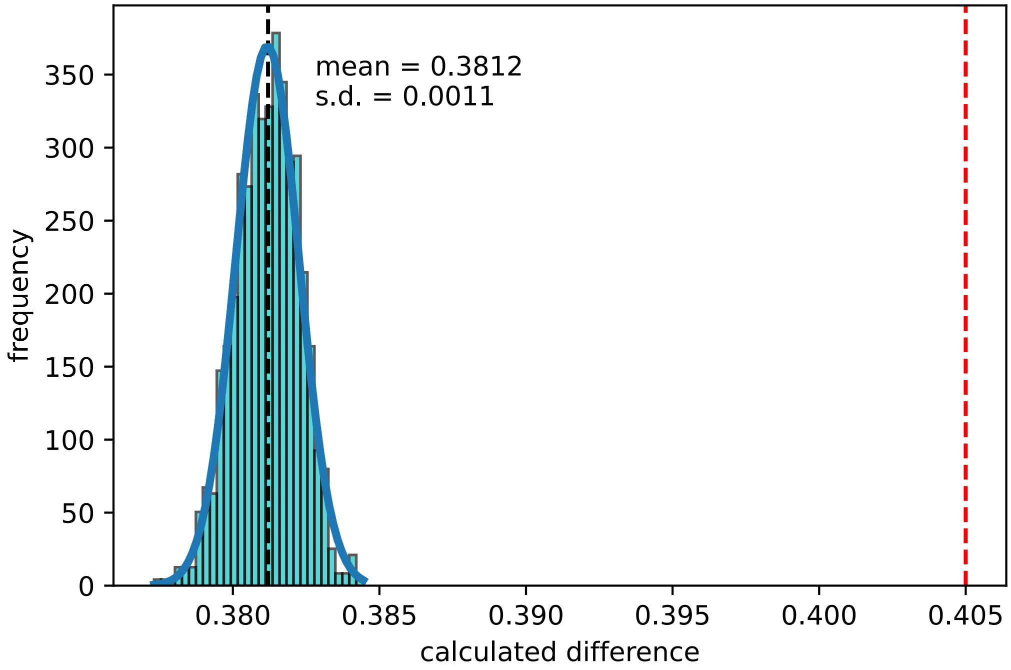

Supplementary Figure S5. The reported normalized difference of the total detrimental mutations occurring in one protein between any two major populations is statistically significantly larger compared to the population of randomly shuffled edgetic profiles. The reported Z-score is ~21 and P-Value < 0.001. We also note that the randomized difference from 1,000 experiments falls within a very tight range.
